# A pre-registered, multi-lab non-replication of the action-sentence compatibility effect (ACE)

**DOI:** 10.3758/s13423-021-01927-8

**Published:** 2021-11-09

**Authors:** Richard D. Morey, Michael P. Kaschak, Antonio M. Díez-Álamo, Arthur M. Glenberg, Rolf A. Zwaan, Daniël Lakens, Agustín Ibáñez, Adolfo García, Claudia Gianelli, John L. Jones, Julie Madden, Florencia Alifano, Benjamin Bergen, Nicholas G. Bloxsom, Daniel N. Bub, Zhenguang G. Cai, Christopher R. Chartier, Anjan Chatterjee, Erin Conwell, Susan Wagner Cook, Joshua D. Davis, Ellen R. K. Evers, Sandrine Girard, Derek Harter, Franziska Hartung, Eduar Herrera, Falk Huettig, Stacey Humphries, Marie Juanchich, Katharina Kühne, Shulan Lu, Tom Lynes, Michael E. J. Masson, Markus Ostarek, Sebastiaan Pessers, Rebecca Reglin, Sara Steegen, Erik D. Thiessen, Laura E. Thomas, Sean Trott, Joachim Vandekerckhove, Wolf Vanpaemel, Maria Vlachou, Kristina Williams, Noam Ziv-Crispel

**Affiliations:** 1 Cardiff University, Cardiff, UK; 2 Florida State University, Tallahassee, FL USA; 3 INICO, Universidad de Salamanca, Salamanca, Spain; 4 Arizona State University, Tempe, AZ USA; 5 Erasmus University Rotterdam, Rotterdam, Netherlands; 6 School of Innovation Science, Eindhoven University of Technology, Eindhoven, Netherlands; 7 National Scientific and Technical Research Council (CONICET), Buenos Aires, Argentina; 8 Cognitive Neuroscience Center, Universidad de San Andrés, Buenos Aires, Argentina; 9 Global Brain Health Institute, University of San Francisco, San Francisco, CA USA; 10 Latin American Brain Health Institute (BrainLat), Universidad Adolfo Ibáñez, Santiago, Chile; 11 Global Brain Health Institute, Trinity College Dublin, Dublin, Ireland; 12 National University of Cuyo, Mendoza, Argentina; 13 DepartameLingüística y Literatura, Facultad de Humanidades, Universidad de Santiago de Chile, Santiago, Chile; 14 University of Potsdam, Potsdam, Germany; 15 Scuola Universitaria Superiore IUSS, Pavia, Italy; 16 University of Tennessee at Chattanooga, Chattanooga, TN USA; 17 University of California – San Diego, San Diego, CA USA; 18 Ashland University, Ashland, OH USA; 19 University of Victoria, Victoria, BC Canada; 20 The Chinese University of Hong Kong, Hong Kong, Hong Kong; 21 University of East Anglia, Norwich, UK; 22 Center for Cognitive Neuroscience, University of Pennsylvania, Philadelphia, PA USA; 23 North Dakota State University, Fargo, ND USA; 24 University of Iowa, Iowa City, IA USA; 25 University of California – Berkeley, Haas, Berkeley, CA USA; 26 Carnegie Mellon University, Pittsburgh, PA USA; 27 Texas A&M University – Commerce, Commerce, TX USA; 28 Departamento de Estudios Psicológicos, Universidad ICESI, Cali, Colombia; 29 Max Planck Institute for Psycholinguistics, Nijmegen, The Netherlands; 30 Centre for Language Studies, Radboud University, Nijmegen, the Netherlands; 31 University of Essex, Colchester, UK; 32 KU Leuven, Leuven, Belgium; 33 University of California – Irvine, Irvine, CA USA; 34 BehavioralSight, Chicago, IL USA

**Keywords:** Embodied cognition, Action-sentence compatibility effect

## Abstract

The Action-sentence Compatibility Effect (ACE) is a well-known demonstration of the role of motor activity in the comprehension of language. Participants are asked to make sensibility judgments on sentences by producing movements toward the body or away from the body. The ACE is the finding that movements are faster when the direction of the movement (e.g., *toward*) matches the direction of the action in the to-be-judged sentence (e.g., *Art gave you the pen* describes action toward you). We report on a pre-registered, multi-lab replication of one version of the ACE. The results show that none of the 18 labs involved in the study observed a reliable ACE, and that the meta-analytic estimate of the size of the ACE was essentially zero.

## Introduction

Embodied approaches to language comprehension are based on the idea that linguistic meaning is grounded in our bodies’ systems of perception, action planning, and emotion. The comprehension of a sentence such as *Meghan served Michael the volleyball* might therefore involve the use of the motor system to internally simulate the actions involved in playing volleyball, the use of the perceptual system to simulate the sights and sounds associated with the sport, and the use of the emotional system to simulate the thrill of the game. This view, which we call the *sensorimotor simulation* view, has received a good deal of empirical support. Behavioral studies suggest a role for motor activity (e.g., Bub & Masson, 2010; Glenberg & Kaschak, 2002; Zwaan & Taylor, 2006), perceptual information (e.g., Kaschak et al., 2005; Meteyard et al., 2007; Stanfield & Zwaan, 2001), and emotional systems (e.g., Havas et al., 2010) in the comprehension process. Neuroscientific evidence for motor simulation comes both from EEG studies showing motor potentials (e.g., Aravena et al., 2010) and mu-rhythm suppression (e.g., Moreno et al., 2015; van Elk et al., 2010) during comprehension, and from fMRI (e.g., Hauk et al., 2004; Huth et al., 2016) and MEG (e.g., García et al., 2019) studies showing motor system activity during language processing. The sensorimotor simulation account is thus supported by converging evidence from a range of methodologies (though see Mahon, 2015, and Mahon & Caramazza, 2008, for an alternative perspective on these data).

**Fig. 1:**
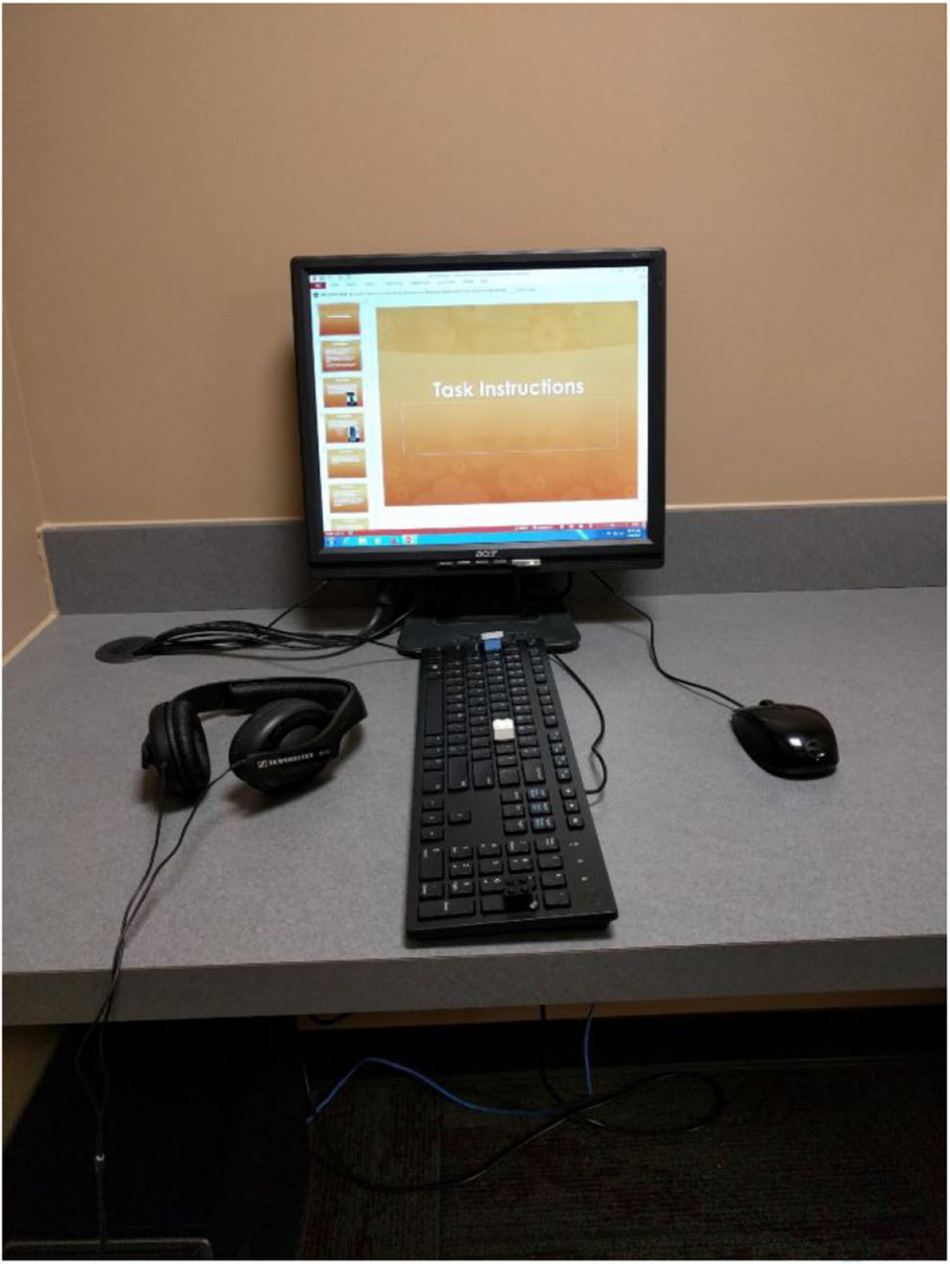
Keyboard configuration for the sensibility judgment task. The central button (white) is held down to initiate the presentation of a sentence. Participants make the sensibility judgment by releasing the white button and moving to the grey button near the monitor (action away from the body) or the black button at the edge of the keyboard nearest the participant (action toward the body)

A well-known effect in the embodiment literature is the Action-sentence Compatibility Effect (ACE; Glenberg & Kaschak, 2002). The ACE is a demonstration that the motor system plays a role in the comprehension of sentences describing particular kinds of action. In the typical ACE paradigm, participants read or hear sentences about sentences that describe action toward (*Art handed you the pen*) or away from (*You handed the pen to Art*) their bodies. Participants are asked to indicate whether the sentences make sense or not. They make this sensibility judgment by executing a motor response toward or away from their bodies. Figure 1 depicts a standard physical set-up for the experiment. Participants press and hold the central (white) button to initiate the presentation of a sentence on the computer screen. To indicate that the sentence makes sense, they release the central button and press either the black (action toward the body) or grey (action away from the body) response button. The canonical ACE is a statistical interaction, where the response times (RTs) are faster when the direction of action for the sentence and the judgment match (a *toward* sentence and a *toward* response, or an *away* sentence and an *away* response) than when the direction of action for the sentence and judgment mismatch (e.g., a *toward* sentence and an *away* response, or an *away* sentence and a *toward* response). A broad interpretation of the ACE is that it reflects priming within the motor system. For example, comprehension of a sentence about action toward your body generates an internal simulation of that action. The internal simulation of the *toward* action in turn facilitates the preparation and execution of a motor response toward the body, and conflicts with the preparation and execution of a motor response away from the body.

The ACE is one of the earliest action compatibility effects reported in the embodiment literature. Evidence for the ACE in these initial studies was generally, but not exclusively, strong. Glenberg and Kaschak’s (2002) Experiment 1 (*n* = 35) found an ACE interaction (faster RTs when the direction of the sentence and the direction of the action match than when they mismatch) of 155 ms, against an average RT of about 1,766 ms, for actions involving transfer of concrete objects. This amounts to just less than a 9% effect in RT. Likewise, in Experiment 2a, Glenberg and Kaschak found an ACE interaction of 170 ms against an average RT of 1,871 ms; again, a 9% RT effect. This observed 9% effect is similar in magnitude to well-established semantic priming effects (see, for instance, Meyer & Schvaneveldt, 1971).

The ACE was subsequently replicated and extended in a number of studies (e.g., Bub & Masson, 2010; Glenberg et al., 2008; Kaschak & Borreggine, 2008; Masson et al., 2008; Taylor & Zwaan, 2008; Zwaan & Taylor, 2006; see García & Ibáñez, 2016, for a review). These studies have helped to clarify the circumstances under which motor activity might be observed during language comprehension (see García & Ibáñez, 2016, for an overview). For example, it has been shown that particular kinds of motor activity can be observed when processing verbs (e.g., Zwaan & Taylor, 2006) and nouns (e.g., Masson et al., 2008). It has also been shown that the magnitude of motor compatibility effects is affected by the timing of the motor response during the processing of language (e.g., Borreggine & Kaschak, 2006; de Vega et al., 2013). Currently, however, there is reason to question whether effects such as the ACE can be observed reliably. Papesh (2015) reports nine experiments aimed at producing the ACE, eight of which resulted in replication failures. Although a number of the experiments in the paper included methodological features that may have hindered the ability to replicate the effect (e.g., visual displays that made the results of the response action ambiguous between the toward/away axis and the up/down axis), the final two experiments in the paper are close replications of the Glenberg and Kaschak (2002) paradigm, and yet fail to show any hint of an ACE. Furthermore, several of the researchers involved in the current replication effort know about unpublished failures to replicate the ACE from other labs, or have unpublished studies in which they failed to produce the effect themselves.

Research paradigms that examine motor compatibility effects are important for both theoretical and practical reasons. On the theoretical side, these paradigms provide an important testing ground for embodied approaches to language comprehension. On the practical side, most of the paradigms are simple, and thus represent a broadly accessible tool for researchers to use to explore the role of the motor system in the comprehension process. As such, it is important to know the extent to which the observed action compatibility effects are replicable, and the extent to which particular specific methods can be used to reliably produce the effects.

Although the question of whether motor compatibility effects are reliable is important, it is also a question that is not straightforward to address. There are many methods for eliciting motor compatibility effects, and these methods differ in a number of important ways. For example, where Glenberg and Kaschak (2002) examined motor effects in responses to whole sentences, and therefore represent a slightly “offline” measure of motor activity, Zwaan and Taylor (2006) found motor effects on specific words during online sentence processing. As a first step in assessing the replicability of motor compatibility effects, we conducted a pre-registered, multi-lab replication of the ACE. We used an adapted version of the original ACE paradigm (Glenberg & Kaschak, 2002) that was used in Borreggine and Kaschak (2006). The choice of this particular version of the ACE paradigm was somewhat arbitrary. We had no strong a priori reasons to expect that one paradigm or the other would produce a stronger or more reliable ACE. Ultimately, we chose the Borreggine and Kaschak (2006) paradigm over the original Glenberg and Kaschak (2002) paradigm in part because we sensed that this paradigm would be slightly easier to execute across a large group of labs.

### Method

#### Pre-registration

The ACE replication project was pre-registered on the Open Science Framework (OSF; https://osf.io/ynbwu/). The pre-registration documents can be found with the following links: initial pre-registration (https://osf.io/356aj/), addendum to the pre-registration (to update details about the data analysis; https://osf.io/8dpyu/), and the pre-registered code for the analyses (https://osf.io/2f3zm/). We direct readers to the project wiki (https://osf.io/ynbwu/wiki/home/) for a brief overview of the project, and direct links to the pre-registration documents, data files, and documents from the project. The materials, methods, data, and code needed to conduct the analyses are all available on the OSF website. We did not deviate from our pre-registered protocol, unless noted otherwise.

#### Lab recruitment

Labs were recruited to participate in the replication project in two phases. In the first phase, specific researchers with (a) previously published work in embodiment or language processing, or (b) expressed interest and/or experience in replication projects were invited to participate. In the second phase, a public call for participation in the replication effort was put out via Twitter. We received commitments to conduct the replication with native English speakers from 14 labs. Due to technical difficulties (one lab) and the inability to recruit enough native English speakers (one lab), the number of labs with native English speakers in this study ended up at 12. We also received commitments to conduct the replication with non-native, but highly proficient, English speakers from six labs. When each lab committed to participate in the project, they were asked to specify a sample size between 60 and 120 participants (in multiples of four, to accommodate the balancing of the four counterbalanced lists used in the experiment; see pre-registered sample sizes at https://osf.io/je7r5/; for details about the settings of individual labs, see https://osf.io/pytrf/). We allowed for variability in each lab’s sample size because (a) we wanted labs to commit to a sample size that was feasible, (b) our primary interest was in the magnitude of the ACE across studies, rather than in the magnitude of the effect in any one study, and (c) sample sizes within the specified range would be as large or larger than the sample sizes typically seen in studies of motor compatibility effects. The sample size recruited by each lab and the number of participants excluded from each lab’s dataset (see elsewhere for screening information) are available on OSF (downloadable at https://osf.io/fmt2k/; under *Sample descriptives*).^1^

#### Participants

The participants were right-handed, native English-speaking (or non-native speakers of English with high proficiency) undergraduate students drawn from the participant pool typically used by each research team’s lab. Handedness was evaluated by administering the Edinburgh Handedness Inventory (Oldfield, 1971), with participants who received a score greater than 0 considered to be right-handed. Across labs, a total of 1,492 participants were recruited for the study. After the aforementioned exclusions were applied (see below for details), 214 participants were eliminated, leaving a sample size of 1,278. Table 1 shows the number of participants across labs before and after elimination, by lab type.

**Table 1:** Sample size characteristics by lab type

	# Labs	Total N	Mean	SD	Min.	Q25%	Median	Q75%	Max.
Native English									
Before Screening	12	942	78.5	23.53	59	60.75	73.5	78.25	132
After Screening	12	903	75.25	22.02	55	60	69.5	73.75	120
Lost			3.25	4.14	0	0	0.5	6.25	12
% Lost			3.91	4.58	0	0	0.8	9.12	10
Non-native English									
Before Screening	6	550	91.67	27.05	60	72.75	86	116.5	123
After Screening	6	375	62.5	31.2	16	44	66	88	95
Lost			29.17	10.11	13	27.25	28.5	32.75	44
% Lost			35.34	21.27	17	22.97	25.7	42.38	73

*Note:* # Labs = number of labs in each category (Native or Non-native English); Total N = total sample size in each category; Mean = average sample size for labs within each category; SD = standard deviation of sample size; Min. = smallest sample size within the category; Q25% = sample size at the 25% quartile; Median = median sample size; Q75% = sample size at the 75% quartile; Max. = maximum sample size within the category

#### Materials

The critical sentences from Borreggine and Kaschak (2006) were recorded by a female speaker. The sound files were trimmed so that there was a minimal amount of silence before the beginning and after the end of the sentence. The files were trimmed using Audacity (Audacity Team, 2015). Eighty sentences were recorded for the experiment. There were 40 critical items (each sentence having a *toward* and *away* version: *Art handed you the pen* (toward) and *You handed Art the pen* (away)), and 40 non-sensible filler items that contained an error (e.g., *Art the pen handed you*). Sentence lengths ranged from 1,022 to 2,065 ms (mean = 1,501.09 ms; standard deviation = 211.68 ms). The complete list of experiment materials is available at https://osf.io/mha5w/.

#### Procedure

Prior to beginning the experiment, informed consent was obtained from each participant. Informed consent procedures were handled in accordance with the procedures determined by each participating lab’s institution.

Participants sat at a computer that had its keyboard oriented perpendicular to their shoulders, with the number pad closest to the body, and the escape key farthest from the body (see Fig. 1). Participants used three keys for their responses: the P key (this was the START key, with a white label), the Tab key (covered with a grey label), and the “+” key (covered with a black label). A picture of the keyboard set-up for each participating lab can be seen on the project’s OSF website (see links to individual lab set-up pictures in the *Keyboard set-up pictures* folder on this page: https://osf.io/ynbwu/files/ ).

Participants first viewed a Powerpoint presentation that explained the task instructions (https://osf.io/vrp3f/). Participants were told that they would be making sensibility judgments about a series of sentences they listened to through headphones. They were instructed to hold down the START button to initiate each trial. As they pressed the START button and the sentence began to play, a grey or black square appeared on the computer screen. If the participant thought that the sentence made sense, the participant released the START button and pressed either the grey or the black response key, depending on whether the grey or black square was on the computer screen. If the participant thought that the sentence did not make sense, the participant continued holding the START button until the trial timed out. At that point, participants would release the START button, and then press it again to initiate the next trial. Once the participants completed the Powerpoint slide show, the experimenter asked them a series of questions to ensure they understood the task instructions.

Once the participants completed the Powerpoint presentation and answered the experimenter’s questions, they started the experiment. The experiment was programmed in E-Prime 2.0 (Psychology Software Tools, Inc., 2016) and included a training phase, an experimental phase, and a few sociodemographic questions. This was followed by a handedness survey that was either administered online or with paper and pencil. Participants were randomly assigned to one of four counterbalanced lists, with the constraint that an equal number of participants be assigned to each list. These lists counterbalanced the direction of action of the critical items (*toward* vs. *away* version of each sentence) with the direction of the response required for that sentence (*toward* or *away* motor response), such that across lists each sentence appeared equally often in the four cells of our design (*toward*/*away* sentence crossed with *toward*/*away* response). The experiment began with four response practice trials, where participants saw the black or grey square appear on the screen and had to press the appropriate response key. Next, there were 18 practice trials in which participants responded to nine sensible and nine non-sensible sentences with feedback, which led seamlessly to the 80 experimental trials (such that the participants did not notice the transition). The items for the experimental trials were presented in a different random order for each participant (see https://osf.io/hf5x2/ to view the E-Prime file).

At the conclusion of the experiment, participants were asked what they thought the experiment was about, and whether they had ever heard of the ACE or any related effects (i.e., if they had learned about the effects in class, or had learned about the effect from participating in another study).

#### Predictions

Our experiment yielded three dependent measures: lift-off time (the time from the beginning of the sentence until participants lifted their finger off the START button to initiate their response), movement time (the time from the release of the START button until the pressing of one of the response keys), and response accuracy. The ACE is the effect of interaction between Sentence Direction and Response Direction (faster responses when the direction of the sentence and the direction of the response match than when they mismatch) on lift-off time (Glenberg & Kaschak, 2002). The effect has typically been observed on the lift-off time measure, and for this reason the critical result for demonstrating a replication of the ACE is the observation of a Sentence Direction by Response Direction interaction on this measure. We established and pre-registered^2^ ranges of effects on RT that we would deem (a) *uninteresting and inconsistent with the ACE theory*: less than 50 ms. Because we decided to analyze the logarithm of RT and RT effects are often changes in scale, we translated this 50-ms effect into a 2.5% effect against a 2s average RT; (b) *consistent with ACE but inconsistent with previous ACE literature*: between 50 ms and 100 ms, or a 2.5–5% against 2 s average RTs; and (c) *consistent with ACE theory and literature*: greater than 100 ms, or 5% against 2 s average RTs. We use equivalence testing (Wellek, 2003) to assess whether the observed ACE was significantly smaller than the stated thresholds. That is, in addition to assessing whether the ACE was reliably different than a null hypothesis of 0 (as is traditionally done in null hypothesis statistical testing), we also tested the observed ACE against the 2.5% and 5% effect values to assess whether the observed effect was to be considered uninteresting (<2.5%), present but smaller than the ACE reported in the literature (ACE between 2.5% and 5%), or present and of the same magnitude as reported in the literature (>5%).

Based on the preceding literature, we did not expect to observe a Sentence Direction by Response Direction interaction on the movement times, or on the accuracy measure. Nonetheless, we acknowledge the possibility that such effects emerge. These effects may be supportive of the general idea of the ACE (i.e., that linguistic and motor processes interact), but such effects are deviations from the canonical pattern of behavior in this paradigm.

### Results

The raw data (https://osf.io/4dru9/) and code for cleaning and analyzing the data (https://osf.io/2f3zm/) are available on the OSF.

Data preparation was performed as described in the pre-registration document.^3^ Left-handed participants, participants who did not complete all trials, participants whose accuracy was lower than 75%, participants who failed to follow task instructions, and non-native English participants whose self-reported competence in oral or written comprehension, or oral or written production fell below 4 (out of 7) on the L2 Language History Questionnaire 2.0 (Li et al., 2014) were excluded from the study. Of the 1,492 participants, 214 (14.34%) were eliminated (54 left-handers, 19 excluded for proficiency, and 141 excluded due to high error rate; no participants were eliminated due to a failure to follow instructions), leaving N = 1,278 participants. Additionally, items were removed from an individual lab’s data when error rates for that item were greater than 15% in the lab.^4^ Finally, individual trials with unusually long or short RTs were eliminated (lift-off latency <1 s, movement times <100 ms or >2,000 ms, or more than 2 SDs from the participant’s mean lift-off time or movement time in a particular condition). Of the 38,993 total trials left after participant and item filtering, 3,287 were eliminated (8.43%), leaving 35,706 total trials across the remaining 1,278 participants. Because the results from native-English speaking countries were collected in conditions closest to the original ones in which the ACE was found, we first present the results from these labs. We then present the results from the non-native English-speaking countries.

#### Native English speakers

Mean values for the three dependent measures (accuracy, lift-off time, and movement time) across the main experimental conditions are presented in Table 2.

**Table 2:** Mean accuracy, lift-off times and move times for native English speakers (SDs in parentheses)

Sentence Direction:	Toward		Away	
Response Direction:	Toward	Away	Toward	Away
Accuracy	.968 (.07)	.965 (.07)	.972 (.07)	.963 (.08)
Lift-off Times	1,928 (192)	1,929 (188)	1,941 (182)	1,942 (179)
Move Times	355 (101)	328 (96)	353 (100)	327 (95)

As expected, accuracy was high overall (96.74%). A logistic mixed model analysis with cue and sentence direction as fixed effects and random intercepts of participant, lab, item, and counterbalance list^5^^,^^6^ suggested that participants were slightly more accurate when the sentence direction was away from them rather than towards them (OR = 1.263, *z* = 2.475, *p* = 0.013, CI_95%_: [1.05, 1.52]). Participants were also slightly less accurate when both the cue and sentence were away from the participant (OR = 0.773, *z* = −2.02, *p* = 0.043, CI_95%_: [0.603, 0.992]). As can be seen in Table 1, these differences are small; they do not threaten the analysis of the RTs. Accuracy was high, suggesting participants took care in the experiment and any speed-accuracy tradeoff is minimal.

**Fig. 2:**
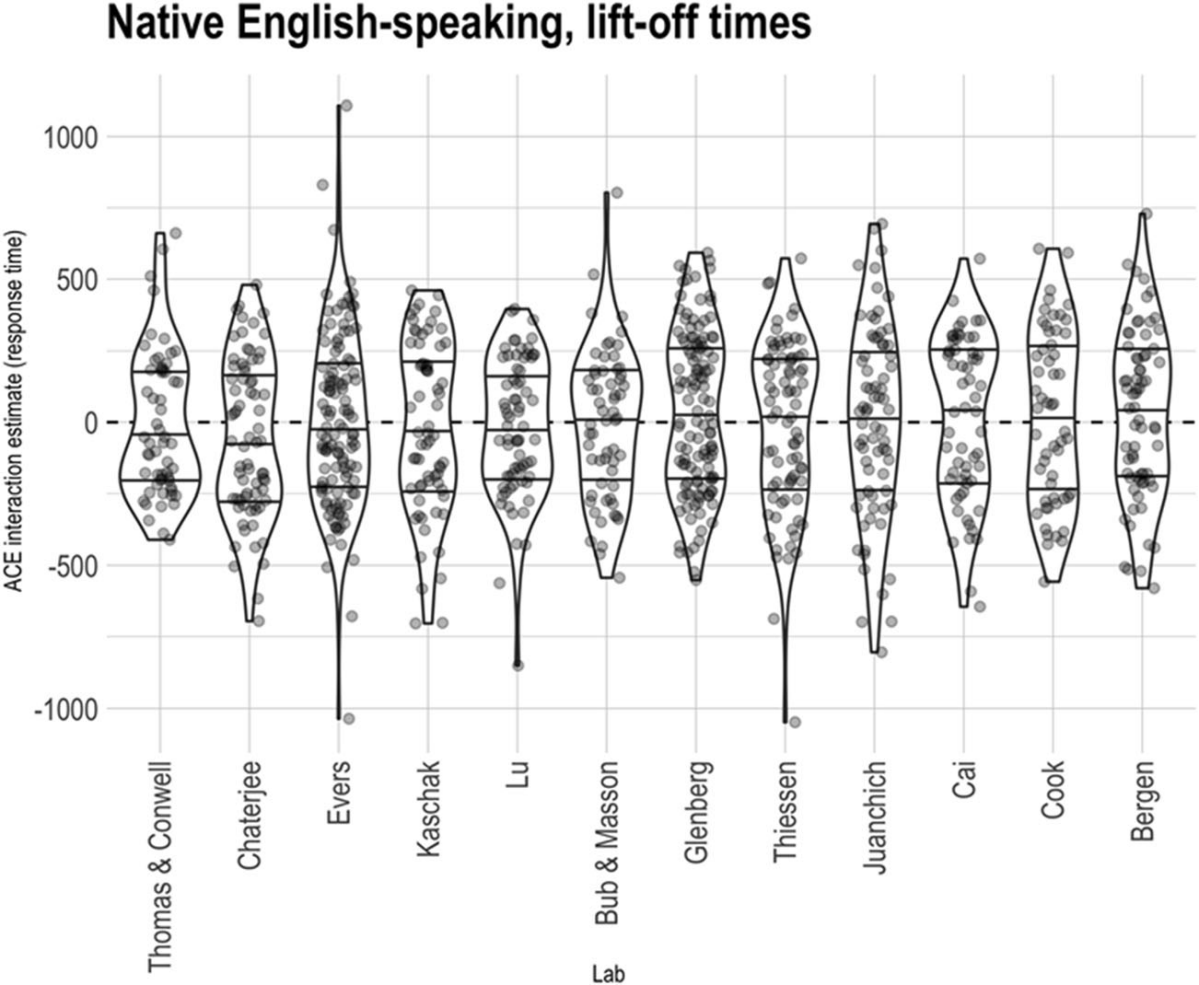
Participant-level Action-sentence Compatibility Effect (ACE) interaction on lift-off time across native English-speaking labs. Within each lab, the middle horizontal line indicates the median effect, and the two other lines indicate the interquartile range. Points are translucent, meaning that darker areas indicate overlapping points and thus higher density

The key predicted ACE interaction is that between cue direction and sentence direction on lift-off times. Figure 2 shows the estimated ACE interaction for all participants in all labs. As can be seen, the median ACE interactions are close to 0 and all within the range that we pre-specified as negligible and inconsistent with the existing ACE literature (<100 ms).

##### Lift-off times

To test the ACE interaction, we fit a linear mixed effects model using the logarithm of lift-off time as the dependent variable, including fixed effects of cue direction, sentence direction, and their interaction, and random intercepts of participant and item. Random intercepts of lab and counterbalance list, as well as variances of random slopes for participants, were estimated to be close to 0 and produced a singular model fit; hence, none of these random effects were included in the analysis.^7^ Consistent with Fig. 2 (see also Fig. 6, right), the average ACE^8^ on the logarithm of the lift-off times was close to 0 (CI_95%_: [-0.006, 0.004]). This corresponds to an average effect on lift-off times of about plus or minus half a percent. The pre-registered equivalence test of (non-)negligibility was significant at *α* = 0.025, as indicated by the fact that the upper end of the 95% CI is within the pre-registered negligible range. The average ACE was not significantly different from 0 at traditional α levels (*F* = 0.121, *p* = 0.728, average *d* = 0.0036).^9^ The data suggest a small and unexpected main effect of sentence direction (*F* = 25.345, *p* < 0.001, CI_95%_: [ -0.010, 0.002]; participants were faster when sentence direction was toward them)^10^ and an effect of cue direction that just barely rises to significance at *α* = .05, *F* = 4.118, *p* = 0.042, CI_95%_: [ -0.006, 0.001]^11^; (although see Díez-Álamo et al., 2020, for five experiments, conducted in Spanish, that replicate the sentence direction effect for both reading times and three types of memory tests. They propose that the effect reflects the importance of objects approaching the body).

**Fig. 3:**
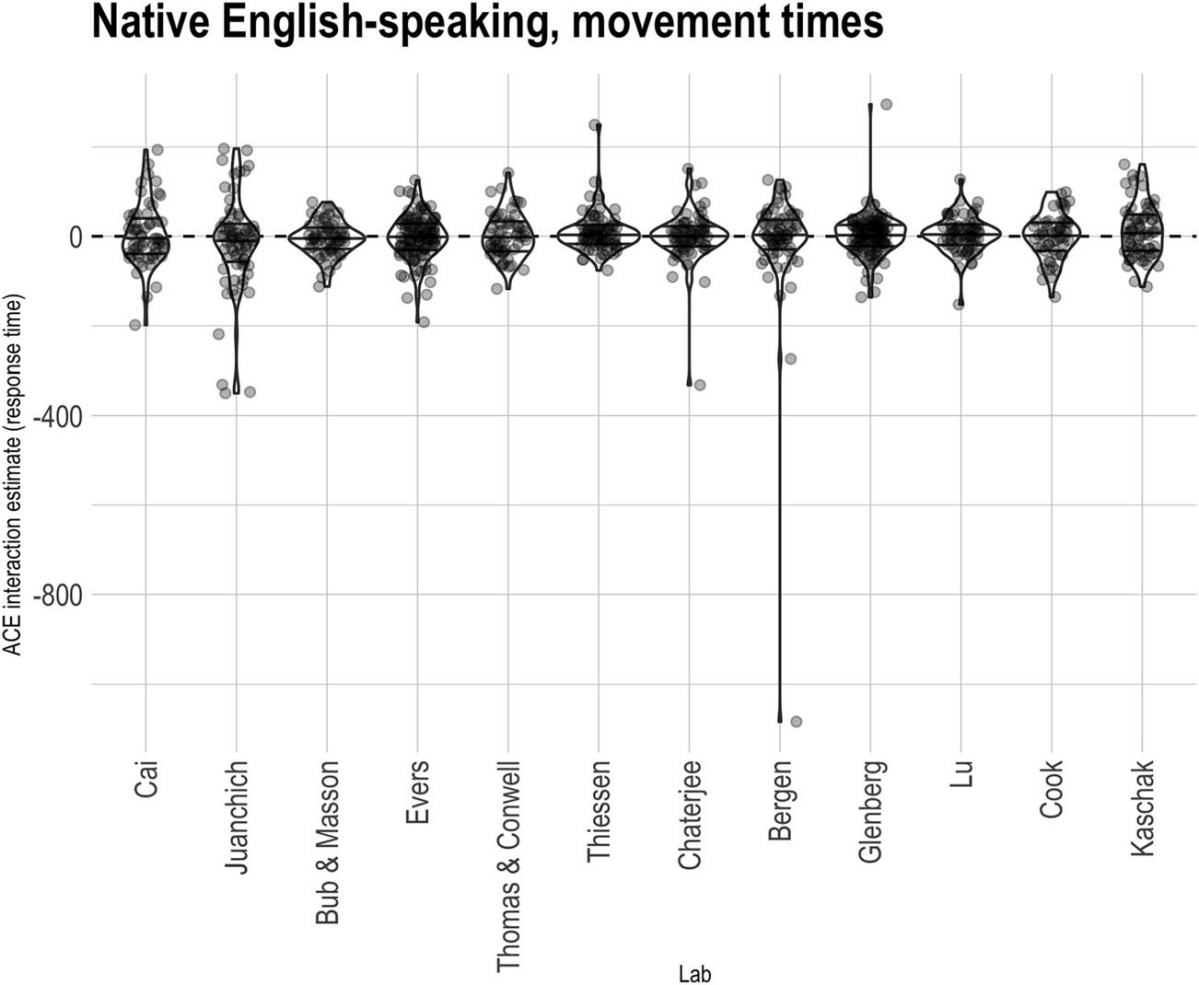
Participant-level Action-sentence Compatibility Effect (ACE) interaction on move time across native English-speaking labs. Within each lab, the middle horizontal line indicates the median effect, and the two other lines indicate the interquartile range. Points are translucent, meaning that darker areas indicate overlapping points and thus higher density

*Movement times.* An ACE interaction was not predicted for movement time. We nevertheless report the pre-registered analysis on movement times for completeness. To test the ACE interaction on movement times, we fit a linear mixed effects model using the logarithm of movement time as the dependent variable, including fixed effects of cue direction, sentence direction, and their interaction, and random intercepts of participant, item, and lab. Random intercepts for counterbalance list, as well as variances of random slopes for participants, were estimated to be close to 0 and produced a singular model fit; hence, none of these random effects were included in the analysis.^12^ There was a theoretically uninteresting but large effect of cue direction such that participants were faster to move when the cued response was away from them (approximately an 8% speeding; *F*>1000, *p <* .0001, CI_95%_: [0.068 0.080]), as well as an effect of sentence direction such that participants were about 0.3% faster to respond to sentences with implied motion toward them (*F=*5.656*, p=* 0.017, CI_95%_: [ -0.002, 0.010]).^13^ However, there was no evidence of an overall ACE interaction on movement times. Consistent with Fig. 3, the average ACE on the logarithm of the movement times was close to 0 (*F=0.509, p = 0.475,* CI_95%_: [ -0.006, 0.012], average *d* = .012). The estimated effect corresponds to a speeding of response-compatible RTs of about 0.3%.

In our pre-registration, we had proposed including responses to the post-experimental questions as predictors in the mixed models to determine if participants’ awareness of the effect moderated the ACE interaction. Participants were asked whether they had heard of the action-compatibility effect (Q2; 2% said “yes”); whether they had heard of the idea that language comprehension involves motor simulation (Q3; 25.2% said “yes”); and whether they had heard of embodied cognition (Q4; 13.3% said “yes”). The extremely small average size of the ACE interaction makes potential subgroup effects difficult to interpret and, if they existed, likely to be an error. Nevertheless, for completeness, we conducted analyses in which we assessed whether the ACE interacted with responses to the three responses to the lift-off time model.^14^ None of the interactions of the responses to these questions with the ACE were significant at traditional *α* levels (all *p*s>.48; see the report at https://osf.io/fmt2k/, under “ancillary analyses”).

#### Non-native English speakers

Data filtering was performed for the bilingual group according to the same criteria as for the labs in native English-speaking countries and consistent with the pre-registration. For bilingual labs, however, many more items were removed due to error rates over 15%. In bilingual labs, a median of 16 out of 40 were removed, which is over twice as many items as were removed in monolingual labs (median: seven out of 40). One should therefore interpret the data from the bilingual labs with caution.

Mean values for the three dependent measures (accuracy, lift-off time, and movement time) across the main experimental conditions are presented in Table 3.

**Table 3:** Mean accuracy, lift-off times and move times for native English speakers (SDs in parentheses)

Sentence Direction	Toward		Away	
Response Direction:	Toward	Away	Toward	Away
Accuracy	.970 (.07)	.966 (.08)	.948 (.10)	.951 (.09)
Lift-off Times	2010 (243)	2001 (248)	2050 (245)	2049 (245)
Move Times	331 (100)	298 (87)	332 (102)	300 (84)

Average accuracy was high overall (95.99%). As in the native English-speaking labs, participants were more accurate on average when the sentence direction was toward the participant (OR = 0.621, *z* = −3.174, *p* = 0.002, CI_95%_: [0.462, 0.833]). No cue by sentence direction interaction was apparent (OR = 1.157, *z* = 0.694, *p* = 0.488, CI_95%_: [0.766, 1.749]).

##### Lift-off latencies

**Fig. 4:**
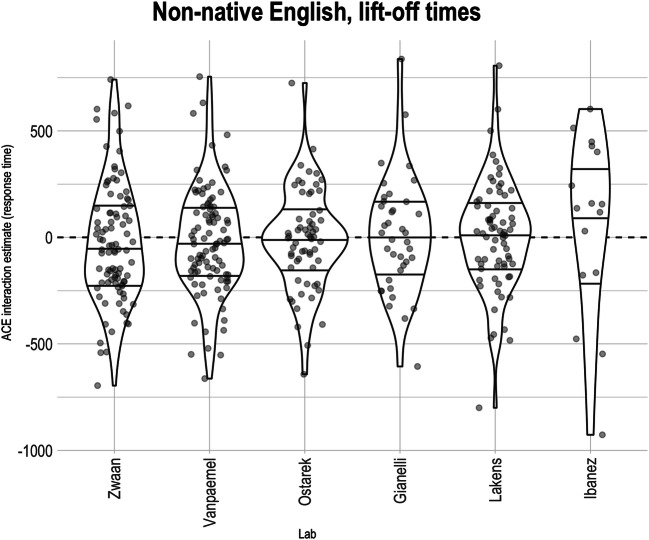
Participant-level Action-sentence Compatibility Effect (ACE) interaction on lift-off times across non-native English-speaking labs. Within each lab, the middle horizontal line indicates the median effect, and the two other lines indicate the interquartile range. Points are translucent, meaning that darker areas indicate overlapping points and thus higher density

Figure 4 shows the estimated ACE interaction for all participants in all bilingual labs. As in the monolingual labs, the median ACE interactions are close to 0 and all within the range we pre-specified as inconsistent with the existing ACE literature (<100 ms).

The test of the ACE interaction proceeded as with the native English-speaking labs. Random intercepts of lab and counterbalance list, as well as variances of random slopes for participants, were estimated to be close to 0 and produced a singular model fit; hence, none of these random effects were included in the analysis. The final linear mixed effects model was thus the same as for the native English speakers. The average ACE effect on the logarithm of the lift-off times was close to 0 (CI_95%_: [−0.012, 0.009]). This corresponds to an average effect on lift-off times of about ± 1%, which is within the bounds we interpret as theoretically negligible. The average ACE effect was not significantly different from 0 (*F* = 0.059, *p* = 0.808, average *d* = -0.019). There appears to be a main effect of sentence direction (*F* = 59.417, *p* < .001, CI_95%_: [ -0.028, -0.013]; participants were faster when sentence direction was toward them^15^), but no evidence of an effect of cue direction, *F* =  0.633, *p* = 0.426, CI_95%_: [-0.005, 0.010] (again, see Díez-Álamo et al., 2020).

##### Movement times

**Fig. 5:**
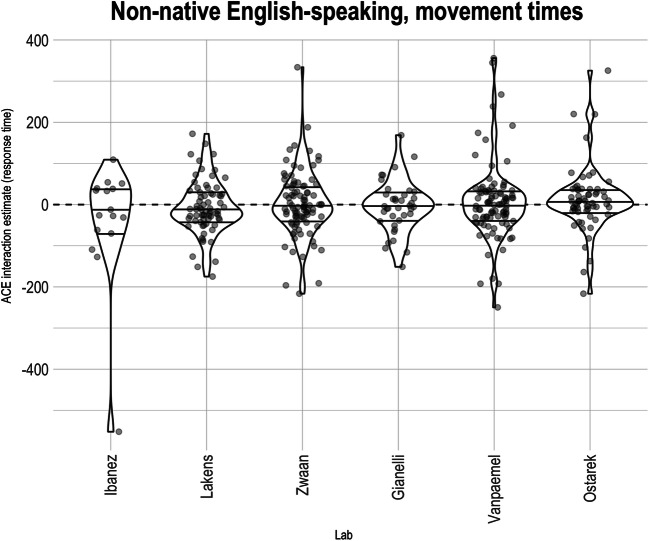
Participant-level Action-sentence Compatibility Effect (ACE) interaction on movement times across non-native English-speaking labs. Within each lab, the middle horizontal line indicates the median effect, and the two other lines indicate the interquartile range. Points are translucent, meaning that darker areas indicate overlapping points and thus higher density

As for the native English-speaking labs, we report the pre-registered analysis on movement times for completeness. Random intercepts for counterbalance list, as well as variances of random slopes for participants, were estimated to be close to 0 and produced a singular model fit; hence, none of these random effects were included in the analysis.^16^ There was a theoretically uninteresting but large effect of cue direction such that participants were faster to move when the cued response was away from them (approximately a 9% speeding; *F* = 469.332, *p <* .0001, CI_95%_: [0.088, 0.112]). The effect of sentence direction was not statistically significant at traditional *α* levels (*F =* 0.286*, p =* 0.593, CI_95%_: [ -0.010, 0.014]). The was also no evidence of an overall ACE interaction on movement times. Consistent with Fig. 5 (see also Fig. 7, right), the average ACE on the logarithm of the movement times was close to 0 (*F =* 0.9715, *p =* 0.324, CI_95%_: [ -0.026, 0.009], average *d* = -0.017). The estimated effect corresponds to a slowing of response-compatible RTs of about 0.8%.

#### Across all labs

**Fig. 6:**
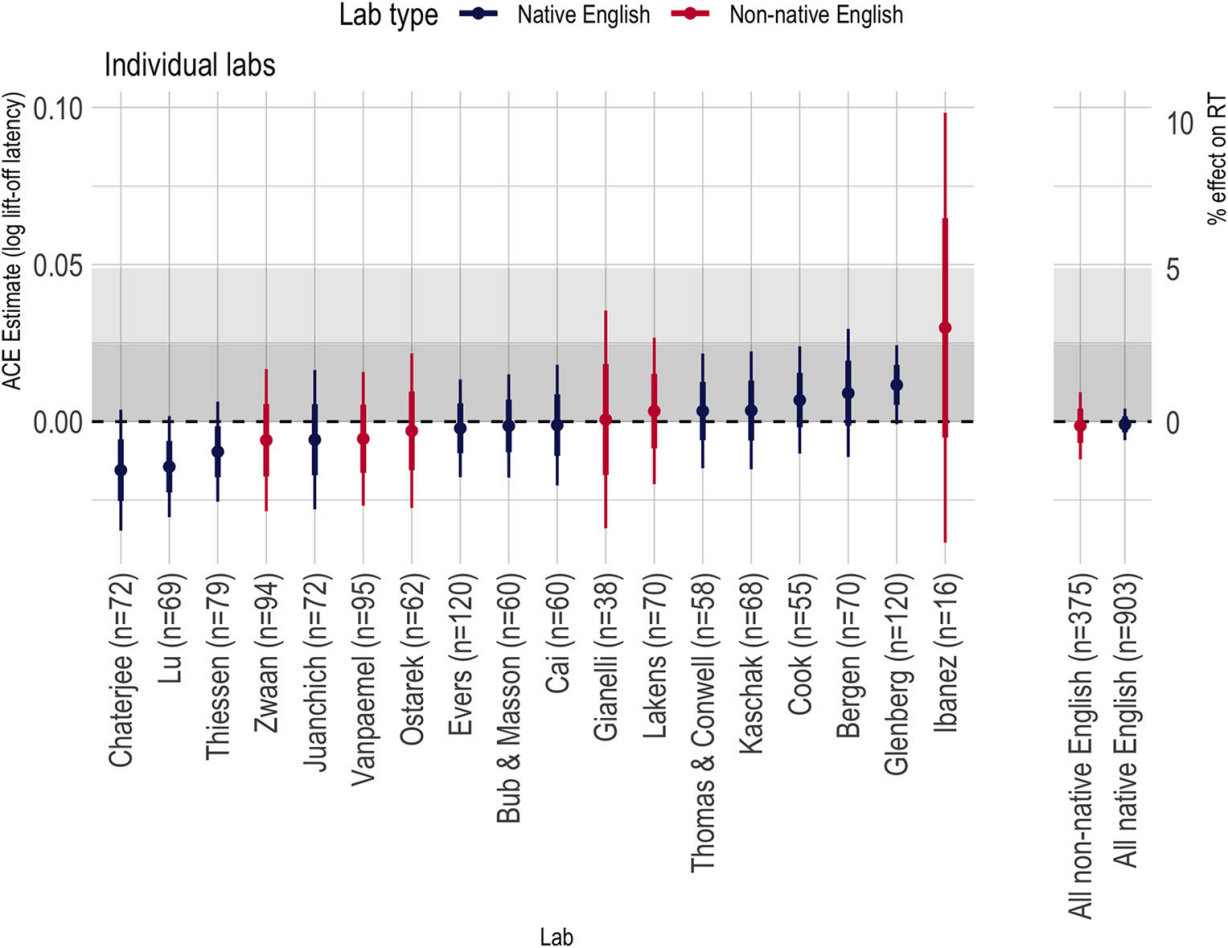
Action-sentence Compatibility Effect (ACE) interaction effects on the logarithm of the lift-off times across all labs. Thick error bars show standard errors from the linear mixed effects model analysis; thin error bars are the corresponding 95% CI. The shaded region represents our pre-registered, predicted conclusions about the ACE: Effects within the lighter shaded region were pre-registered as too small to be consistent with the ACE; effects in the dark gray region were pre-registered as negligibly small. Above the gray region was considered consistent with the extant ACE literature

**Fig. 7:**
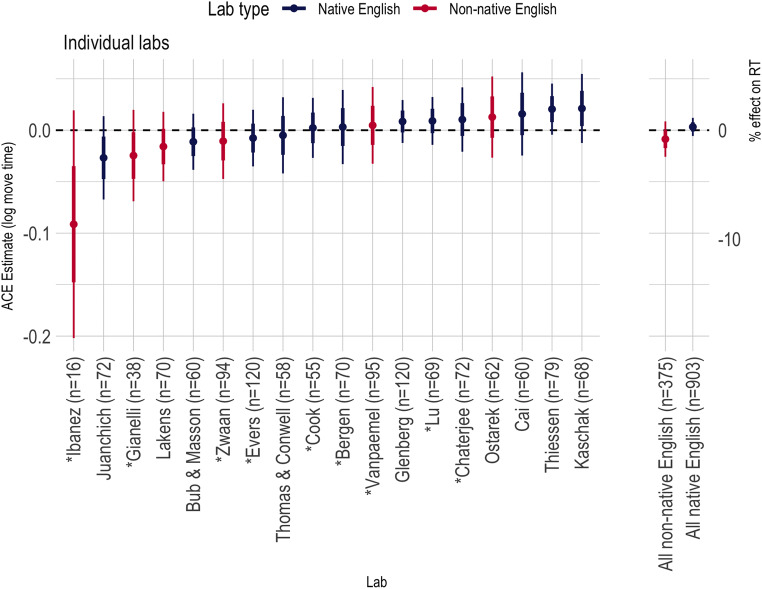
Action-sentence Compatibility Effect (ACE) interaction effects on the logarithm of the move times across all labs. Thick error bars show standard errors from the linear mixed effects model analysis; thin error bars are the corresponding 95% CI. Asterisks before the names indicate a singular fit due to the random effect variance of items being estimated as 0. For comparability of the effect, we include them here so that all effects presented were estimated using the same model

Figures 6 and 7 summarize the ACE in the lift-off times and movement times, respectively, across all labs in this study. Across all labs, the ACE was small and within the range defined as theoretically negligible. No lab showed an ACE effect that rose to traditional levels of statistical significance to either dependent variable.

Figures 6 and 7 show that there is remarkably little heterogeneity across labs, which may be due to the standardized nature of our task. Due to the small numbers of labs (particularly in the non-native-English group) meta-analytic estimates of heterogeneity will be volatile, but we report them for completeness. To calculate *τ*^2^ and *I*^2^, we estimated the ACE effect separately for each lab using the specified linear mixed effects model (minus the random effect of lab, if it was included in the overall model). The effect and the standard error were submitted to the *rma* function in the R package *metaphor* (Viechtbauer, 2010). Table 4 shows estimates of *τ*^2^ and *I*^2^ and their 95% CIs for the ACE interaction on the logarithm of RT.

**Table 4:** Meta-analytic estimates of heterogeneity across labs

	Quantity	Estimate	CI_95%_
Native English			
Lift-off times	*τ*^2^	<0.01	[0.000, 0.0001]
	*I*^2^	19.89%	[0.00%, 63.05%]
Movement times	*τ*^2^	<0.01	[0.000, 0.0003]
	*I*^2^	<0.01%	[0.00%, 56.10%]
Non-native English			
Lift-off times	*τ*^2^	<0.01	[0.000, 0.0002]
	*I*^2^	<0.01%	[0.00%, 54.50%]
Movement times	*τ*^2^	<0.01	[0.000, 0.0060]
	*I*^2^	0.21%	[0.00%, 92.94%]

### Discussion

We undertook a multi-lab, pre-registered replication of the ACE to determine whether the effect could be produced reliably using a standard paradigm in the field. The results of the replication effort are clear: This version of the ACE was not statistically significant in any of the individual studies, and the meta-analytic effect size was close to zero (see Fig. 6).^17^ In the remainder of the paper, we consider the theoretical and practical implications of this result.

The ACE is one of the first action compatibility effects reported in the literature, and the effect is often cited as important empirical support for embodied theories of language comprehension (e.g., Mahon & Caramazza, 2008; Papesh, 2015). Our failure to replicate the ACE undermines the extent to which the published literature in this area might be taken as evidence for embodied cognition. Whereas it might be tempting to conclude that our failure to replicate the ACE deals a critical blow to the embodiment approach, we believe that a more cautious conclusion is in order. Evidence that the motor system plays a role in language comprehension comes from multiple sources. For example, there are behavioral studies employing methods both similar (e.g., Zwaan & Taylor, 2006, with participants using a left or right rotation of the hand to respond, rather than the toward and away actions used here) and dissimilar (e.g., Masson et al., 2008, employ a method where participants are trained to generate specific hand postures; Olmstead et al., 2009, use changes in the oscillation of the arms to detect motor effects during comprehension) to the ACE method used here. There are also neuroimaging studies employing measures such as EEG (e.g., van Elk et al., 2010) and fMRI (e.g., Hauk et al., 2004) that show motor activity during the processing of language. Our results undermine confidence in one of these sources of evidence (behavioral studies similar to the ACE paradigm used here), but do not have clear implications for the other sources of evidence (e.g., the non-ACE behavioral studies; the neuroimaging studies). A full assessment of the theoretical claims of the embodied research program requires a thorough vetting of the reliability of the effects from a range of paradigms.

There are two more practical points that we would like to make. The first is that our results suggest that researchers should be cautious about using the ACE paradigm to study motor compatibility effects. This word of caution applies both to researchers wishing to extend the ACE paradigm to test particular claims about language processing, and to researchers wishing to use the ACE paradigm to generate an individual difference measure of “motor simulation” (or some such concept). The fact that we only used a single ACE paradigm leaves open the question of how broadly this caution should be applied to the range of tasks that have been used to demonstrate or assess motor compatibility effects. Although we cannot provide a definitive answer to this question, it is our sense that researchers interested in pursuing work with this paradigm would benefit from employing transparent practices such as pre-registration so as to increase confidence in the results that are reported.

The second practical issue that we would like to raise concerns the design of experiments aimed at demonstrating the ACE and ACE-like effects. Experiments of this sort tend to use a relatively small set of items for the purpose of having items that provide a sufficient match between the action described in the sentence and the action that participants are asked to generate for their response. It has long been known that experimental items represent an important source of variability within an experiment (e.g., Clark, 1973), and accounting for this variability in appropriate ways is essential for reaching sound conclusions about the nature of the effects that are present. Experiments that have too few items or trials are unlikely to have enough precision to allow researchers to observe effects against the background of the item- (and participant) based noise in the data. Exploratory data analysis showing that item-based variability may drive part of the unreliability of the ACE across experiments is presented on the project OSF site (https://osf.io/x97qg/). To the extent that item-related issues undermine the reliability of the ACE, it suggests that researchers interested in exploring the effect design experiments that use a larger number of items.

The results of our study indicate that this version of the ACE paradigm does not reliably produce the predicted motor compatibility effect. This finding may be legitimately interpreted as an end unto itself (showing that a particular effect is not reliable), but it is our sense that the results of pre-registered replication studies such as ours should also be seen as a beginning – the first step in a broader effort to evaluate the evidence for the role of the motor system in language comprehension, and the circumstances under which such effects might be reliably demonstrated.

## Footnotes

1 The final sample size for the Ibanez lab was much smaller than the sample size of the other labs due to a high rate of participant attrition (principally due to low participant accuracy).

2 The pre-registration document for these ranges of RTs can be found at https://osf.io/8dpyu/.

3 There was one exception: in the pre-registered data-cleaning code, the assessment of extreme RTs for removal was not properly conditioned on accuracy. This error was found and fixed in the cleaning code used for all analyses. The final cleaning was therefore consistent with the text of the pre-registration, but not entirely consistent with the pre-registered code.

4 Several items were removed in all labs, including “You hit Paul the baseball,” a garden-path sentence. Items that were commonly removed tended to be difficult to parse or contain low-frequency words. In contrast, “Liz told you the story,” a more straightforward sentence, was not removed in any lab.

5 In lme4’s model notation: Accuracy ~ CueDirection*SentenceDirection + (1|ptid) + (1|lab) + (1|ItemNumber) + (1|Counterbalance.List)

6 DF > 8138 for all analyses reported in the paper.

7 The final model, in lme4’s model notation, was log(LiftOffLatency) ~ CueDirection*SentenceDirection + (1|ptid) + (1|ItemNumber).

8 All confidence intervals are Wald approximations; due to the large amount of data, other methods struggle. For tests of effects, we used the lmerTest R package with Type II sums of squares to provide p values using the Satterthwaite’s degrees of freedom (Kuznetsova et al., 2017). In order to keep the text readable, we have not reported the degrees of freedom in the text because all estimated denominator degrees of freedom were greater than 8,000.

9 We computed the *d* for each participant by computing the average standard deviation of the response time (about 270 ms for the lift-off latencies, and 53 ms for the move times) for each participant in each condition, and dividing the participant’s ACE effect by this number.

10 This effect disappears when a random slope for sentence direction is added to the item random effect group, so it may be spurious in this data set. See the Online Supplemental Materials at https://osf.io/4zgvp/ for additional non-preregistered linear mixed effects model analyses.

11 Some of the effects that we report as statistically significant are likely to be regarded as negligible, and would not be statistically significant if we implemented an alpha-correction across the large number of tests that were conducted on the data. We report them here for the sake of completeness.

12 The final model, in lme4’s model notation, was log(MoveTime) ~ CueDirection*SentenceDirection + (1|ptid) + (1|ItemNumber) + (1|lab). Note that this is different from the model that was fit to the lift-off times, due to the fact that we performed a separate model-fit checking process for each dependent variable, as preregistered.

13 The CI is based on a Wald approximation. In some cases, the interval for a statistically significant effect may include 0.

14 The model, in lme4’s model notation, was log(LiftOffLatency) ~ CueDirection*SentenceDirection*PostQ2 + CueDirection*SentenceDirection*PostQ3 + CueDirection*SentenceDirection*PostQ4 + (1|ptid) + (1|ItemNumber), where PostQ2, PostQ3, and PostQ4 are the three yes/no questions asked after the experiment.

15 As with the native English-speaking labs, we also fit a non-preregistered analysis where items were allowed to vary with respect to their sentence direction effect. This greatly diminishes the evidence for the fixed effect (*F=6.13, p=0.02*). For details, see the Online Supplementary Material at https://osf.io/4zgvp/.

16 The final model, in lme4’s model notation, was log(MoveTime) ~ CueDirection*SentenceDirection + (1|ptid) + (1|ItemNumber) + (1|lab). As in the English-speaking labs, this is different from the model that was fit to the lift-off times, due to the fact that we performed a separate model-fit checking process for each dependent variable.

17 Statistical power is always a concern when interpreting a null effect. As discussed on the project website on OSF (https://osf.io/fmt2k/), the meta-analysis was sufficiently powered to detect effects of the magnitude that we specified in our pre-registration.
